# On the Effects of Biliary Calculi

**Published:** 1829-10-01

**Authors:** 


### ON THE EFFECTS OF RECENTLY FORMED BILIARY CALCULI. By M. BRICHETEAU, M. D. PROPOSAL OF ICE TO THE EPIGASTRIUM.

Every practitioner of experience, knows the dreadful sufferings occasioned by gall-stones—more especially during the first attacks of this complaint, and before the biliary ducts have become accustomed to the passage of calculi along their internal surfaces. The author of the paper before us, who is a distinguished pathologist, as well as physician, believes he can offer to the public "un moyen plus efficace qu'aucune autre de calmer promptement des accès insupportables qu'éprouvent les malades." A remarkable case, which we here subjoin, was the origin of this communication.

*Case* 1. Madame M——, aged 26 years, had been exposed, since the age of eighteen, to the greatest mental anxiety, in consequence of an ill-judged marriage. In the last seven years she had had four severe attacks of vomiting, the last of which forms the subject of the present case. For a day or two preceding the former attacks the patient experienced acute pains in the back and epigastrium, to which succeeded painful vomiting of yellow and green bile. These pains and vomitings continued for two or three days, after which she completely recovered her usual health. No fever accompanied these attacks. On the 15th July, after the usual premonitory sensations, the attack of bilious vomiting came on, and after lasting the usual time, left a state of indigestion and pains in the epigastrium, which had not been the case after former visitations of the complaint. On the 8th day another bilious vomiting came on, and in the night of that day she had a rigor, with sharp pain in the right hypochondrium. On the succeeding day the eyes and skin were jaundiced—the pulse was quickened—the head ached, &c. Some laudanum was given, which mitigated the pain, but the jaundice became more intense, and no doubt remained that biliary calculi were the cause. On the fourth day of the jaundice a profuse perspiration broke out, and the yellow colour almost entirely disappeared. The urine, however, was much loaded. The pain of side ceased. On the 5th of August, she had another attack of pain in the epigastrium, and vomiting of green bile. On the sixth these symptoms were very distressing, and no ease could be obtained. Eighteen leeches were applied to the anus—and a few drops of laudanum were administered. On the 7th there was a mitigation, and the accession gradually disappeared. But there remained pains in the epigastrium, hypochondrium, and right shoulder. In a few weeks another violent accession of pain and vomiting came on, which was relieved by leeching the right side and the epigastrium. On the 3d of December, fresh paroxysm, more severe than any that preceded, threatened this afflicted patient's life. General icterus followed, as on most of the other attacks, and the fit ended—but only to be renewed in the course of a few days. The pains in the epigastrium and in the back were most excruciating on these occasions. *In the course of January, the attacks began to assume a regular periodicity, returning once a week, and lasting one day each time.* Finding that bleeding, anodynes, and other measures were unattended by any success, M. Bricheteau had recourse to purgatives of salts and senna. The first produced violent pains in the bowels, succeeded by a discharge of numerous small biliary calculi, after which she had no more paroxysms, and her general health became re-established. Nearly two years afterwards this lady experienced another attack of this terrible malady. But M. Bricheteau now determined to try a new remedy—and that was the application of ice to the right hypochondrium. The fit was speedily terminated. Four months after this, a similar accession of the disease returned—and again the application of ice put a speedy end to the paroxysm.

*Case* 2. Madame R——, aged 45 years, had for several years been subject to attacks of severe pain in the epigastrium, obliging her to bend herself nearly double, and make great pressure at the pit of the stomach. These attacks lasted several hours, and were renewed every two or three months. M. Bricheteau was consulted, and tried several remedies, without success. He then ordered a bladder filled with pounded ice to the hypochondrium in one of these attacks. The application itself produced considerable suffering; but by the time the ice in the bladder was dissolved, and the temperature of the water brought to that of the skin, the lady was agreeably surprised to find that the internal pain, by which she had been harrassed for 36 hours, was entirely gone. In some subsequent attacks, the same remedy was found equally efficacious. M. Bricheteau assures us that the attacks of this lady were really those of biliary calculi—or, at all events, of biliary obstruction.

this we are by no means convinced. Be this as it may, let us proceed to the third case.

*Case* 3. A young lady, 26 years of age, had complained for a long time of various pains, which came on periodically, and generally in the epigastric region. The complaint was considered by some as hysteria—by others as biliary calculi. During the attacks she experienced the most excruciating pains in the right hypochondrium, where the slightest pressure could not be endured. Pain was also experienced in the right shoulder, and the knees were kept drawn up to the belly during the paroxysms. Amenorrhœa and great emaciation were added. These paroxysms generally lasted twelve or fifteen hours. In one instance it lasted three days, and the patient nearly lost her life. At last she passed a great number of small biliary calculi, not larger than the heads of pins, after which, she rapidly recovered.

REMARKS. M. Bricheteau assures us that, since the occurrence of the first case which we have quoted, many others have occurred, in which the application of ice to the epigastrium has put a quick termination to the pain and spasms and thus greatly curtailed the duration of the attack. We have had some experience in these painful complaints, and we are well aware of the difficulty of even relieving them, after the paroxysm has once commenced.

We believe that, in many of these cases, gall-stones are suspected when they do not exist. Thus in case 2 of this paper there is no positive evidence of gall-stones. There is no mention of jaundice—the most unequivocal symptom of gall-stones. We do not deny indeed that biliary calculi in the cystic duct, may produce all the phenomena, except jaundice, while the calculi are only thus far advanced. But, if they once get into the ductus communis, jaundice is almost inevitable. We have strong reason, however, to believe—indeed to know, that spasmodic affections of the stomach itself—of the diaphragm—and perhaps of other contiguous parts where muscular fibres enter into the organic structure, are capable of inducing all the phenomena usually seen in the attacks under consideration, excepting jaundice. Nay, we are inclined to think that the yellow tinge of the eyes and skin may be produced by these spasmodic affections, quite independently of biliary calculi. The more important part of the treatment is the prevention—for during the paroxysms we can do little more than exhibit anodynes. The application of ice is a novel item in the methodus medendi, and of this we have no experience. The prevention may almost always be effected by plain and abstemious diet—by regular exercise, and by bitter aperients that merely act once in the 24 hours. By these means we have, in numerous cases, removed the tendency to these periodical attacks, after the habit had been long established, and the general health very much deteriorated.

VIII.

